# Plasmids Increase the Competitive Ability of Plasmid-Bearing Cells Even When Transconjugants Are Poor Donors, as Shown by Computer Simulations

**DOI:** 10.3390/microorganisms11051238

**Published:** 2023-05-08

**Authors:** João S. Rebelo, Célia P. F. Domingues, Teresa Nogueira, Francisco Dionisio

**Affiliations:** 1 cE3c—Centre for Ecology, Evolution and Environmental Changes & CHANGE, Global Change and Sustainability Institute, Faculdade de Ciências, Universidade de Lisboa, 1749-016 Lisboa, Portugal; joaorebelo_4@hotmail.com (J.S.R.); celiapfd@hotmail.com (C.P.F.D.); teresa.nogueira@iniav.pt (T.N.); 2 INIAV—National Institute for Agrarian and Veterinary Research, 2780-157 Oeiras, Portugal

**Keywords:** conjugative plasmid, plasmid fitness-cost, biological weapons, plasmid transfer rate, donor ability, recipient ability, transconjugants, antibiotic resistance genes, virulence genes

## Abstract

Bacterial cells often suffer a fitness cost after conjugative plasmids’ entry because these cells replicate slower than plasmid-free cells. Compensatory mutations may appear after tens of or a few hundred generations, reducing or eliminating this cost. A previous work based on a mathematical model and computer simulations has shown that plasmid-bearing cells already adapted to the plasmid may gain a fitness advantage when plasmids transfer into neighboring plasmid-free cells because these cells are still unadapted to the plasmid. These slow-growing transconjugants use fewer resources, which can benefit donor cells. However, opportunities for compensatory mutations in transconjugants increase if these cells become numerous (through replication or conjugation). Moreover, transconjugants also gain an advantage when transferring the plasmid, but the original donors may be too distant from conjugation events to gain an advantage. To understand which consequence prevails, we performed further computer simulations allowing versus banning transfer from transconjugants. The advantage to donors is higher if transconjugants do not transfer plasmids, mainly when donors are rare and when the plasmid transfer rate (from donors) is high. These results show that conjugative plasmids are efficient biological weapons even if the transconjugant cells are poor plasmid donors. After some time, conjugative plasmids gain other host-benefit genes, such as virulence and drug-resistance.

## 1. Introduction

Almost 5 million deaths yearly are associated with antibiotic resistance worldwide [1,2]. Different causes have been identified, from the microscopic and molecular level to human social behavior and psychological traits [3,4,5]. At the molecular level, conjugative plasmids play a significant role in the spread of antibiotic-resistance genes in general and in clinical settings [6,7]. Therefore, understanding the behavior and ecology of these mobile genetic elements is thus very important combat the antibiotic-resistance pandemic [8].

Bacteria that have recently received a plasmid usually replicate more slowly than before and more slowly than otherwise isogenic plasmid-free cells. For example, DNA entering the recipient cell as single-stranded DNA during plasmid transfer can activate the SOS response halting cell replication [9,10]. Another source of plasmid cost is the interaction between proteins encoded in the plasmid with cellular networks [11]. For a review of these and other identified causes of plasmid costs, see [12]. Therefore, it is a paradox that conjugative plasmids are so common among bacterial populations.

Sometimes plasmids bring a benefit, like virulence or antibiotic resistance genes, but not always. Even if plasmids harbor these genes, they may be useless for long periods. For example, genes conferring resistance to a specific antibiotic are worthless if that drug is not present in the environment. Additionally, plasmid-bearing cells may gain compensatory mutations [13], but they may take too long to occur. Moreover, interactions with other plasmids may also help plasmid maintenance [14,15,16], but again, this implies that one must find mechanisms to ensure the maintenance of the other plasmids.

To solve the paradox of plasmid ubiquity, we have recently proposed the hypothesis that plasmids may act as biological weapons [17]. The first step of this mechanism is that plasmid-bearing cells adapt to each other. This adaptation occurs because compensatory mutations appear on the chromosome after tens or hundreds of generations, reducing and often eliminating the fitness cost of the plasmid [18,19]. If the compensatory mutation is in the chromosome, the plasmid imposes a fitness cost on neighboring recipient cells when it moves to them to form transconjugants. As a result, these cells’ replication rate is lower than before the plasmid was acquired, and they consume fewer resources for tens or hundreds of generations while they fail to adapt to the plasmid. Meanwhile, the donor cells can take advantage by growing faster than the transconjugant cells around them [17].

In that work, we used a mathematical evolutionary model to analyze the microenvironment near transfer events in a structured environment. We found that donor cells would benefit from donating plasmids if D/R were above c/b, where D and R are the numbers of donor and recipient cells near the cell that received the plasmid, where b is the fitness of non-adapted transconjugants, and where c is the plasmid cost in donor cells. (In this context, we considered that two bacteria are in the neighborhood of each other if they compete for resources.) The mathematical model only analyzed the initial conditions. However, plasmid transfer (forming transconjugants), replication of all bacterial types, and, possibly, compensatory mutations occur. Because the conditions may change from those met initially, we also performed several hundred simulations using a wide range of parameter values. For example, the plasmid transfer rate spanned four orders of magnitude; the cost to the transconjugant cell before and after the compensatory mutations appeared were 0%, 40%, and 60%; and the number of generations required for the appearance of compensatory mutations was 70 or 400 generations, as well as four different proportions of donor cells, a total of 288 different conditions. These simulations corroborated our hypothesis. In fact, in many conditions, especially in structured habitats, the success of donor cells is higher compared to a similar system without plasmid transfer (with the same parameters) [17].

However, a second factor (beyond plasmid transfer from donor cells) may confer an advantage to these harmful donor cells. According to our hypothesis, transconjugants may transfer the plasmid to other recipient cells, possibly increasing the benefit to the original plasmid donor population even further. However, the diversity in plasmid transfer rate between bacterial strains and species is high, spanning several orders of magnitude [20,21]. Therefore, the donor ability of transconjugants may be orders of magnitude lower than that of the original plasmid-bearing cells. Indeed, the donor ability of some bacteria can be undetectable, even if cells of other strains of the same species are excellent donors of the same plasmid [20]. If that is the case, the second factor may be negligible if the transfer rate between newly formed transconjugants and other recipients is low.

On the other hand, plasmid transfer from transconjugants may be disadvantageous to the original donors. If transconjugants can transfer the plasmid, more transconjugants form, so there are more opportunities for the appearance of compensatory mutations. Furthermore, unused resources of transconjugants may be valuable to nearby transconjugants and recipient cells but inaccessible to the original donor cells if those transconjugants have formed at sites too distant from the original donor population.

In summary, plasmid transfer from transconjugants can be advantageous or disadvantageous to the original plasmid-bearing population (donor population): advantageous because the plasmid causes more harm to neighboring recipient cells or disadvantageous because this would increase the chances of the appearance of plasmid-adapted bacteria or because transfer events can occur far from the original donor cells. This paper aims to understand which effect prevails. To test this hypothesis, we performed and compared the fitness advantage of the donor population with two different types of simulations: with zero transfers from transconjugants versus transfers from transconjugants with the same donor ability as the original donor cells. We found that if the transfer rate is high and donors are rare, the fitness advantage of the donor population may be higher if transconjugants do not pass the plasmid to other cells.

## 2. Materials and Methods

With this model, we aimed to understand whether plasmid transfer from transconjugant bacteria benefits the original plasmid-bearing population. For that, we adapted a model previously developed by us [17] based on the works published by the research groups of Krone and Top [22,23,24]. These authors conceived a computer model and fine-tuned model parameters so that the density of plasmid-bearing (including the original donors and transconjugants) and recipient cells match experimental results concerning bacterial growth and plasmid transfer in a structured habitat (surface) of 1000 × 1000 sites in a grid with an area corresponding to 1 mm^2^ [22,23,24].

In this new model, we enable or disable the possibility of transconjugants to transfer plasmids and compare the success of donor cells in the two systems. If transconjugants do not transfer plasmids, only donor bacteria conjugate. The model was developed in Python and is available on GitHub (https://github.com/jrebelo27/harmfull_plasmids_no_retransfer accessed on 25 April 2023).

### 2.1. Initial Conditions

The model simulates a grid of 1000 × 1000 spaces (or sites) with periodic boundaries; i.e., the top of the grid is in contact with the bottom, just as the left side connects to the right. Each site can be empty or contain only one bacterium. The simulation starts by randomly distributing 10,000 bacteria that are plasmid-bearing (donors, D) or plasmid-free (recipients, R), in the following ratios: (i) 9900D:100R, (ii) 5000D:5000R, (iii) 100D:9900R, and (iv) 10D:9990R. The donor bacteria have an associated fitness cost *c* (0 or 0.1) since they carry the plasmid.

### 2.2. Flow of the Model

After the initial distribution of bacteria, the computer program chooses a bacterium randomly, and the following processes can occur: growth, conjugation, and segregation. Each bacterium has two types of neighborhoods associated: (i) local neighborhood, defined by the 3 × 3 space centered on itself, and (ii) nutrient neighborhood, defined by the 7 × 7 space centered on itself.

Bacterial growth depends on the existence of an empty site in the local neighborhood of the chosen bacterium and the growth rate. The growth rate (ψ) is given by:

(1) $$\psi (C)=\{\begin{array}{c} \psi^{max},\;if\;C\geq \;\theta \\ \psi^{max}\;\frac{C}{\theta },\;if\;0\leq C<\theta \end{array}$$

In these mathematical expressions, θ is a threshold for growth rate and ψ^max^ = 1 − plasmid cost. In the simulations, we set θ=0.8. *C* represents the available nutrients and corresponds to the proportion of empty sites in the nutrient neighborhood.

If the chosen bacterium is a donor able to grow, it can also transfer the plasmid to a recipient bacterium (bacterial conjugation). This process depends on the existence of a recipient bacterium in the local neighborhood of the donor bacterium, and the conjugation rate (γ) given by:

(2) $$\gamma \left(C\right)=\{\begin{array}{c} \gamma_{max}\;\;\;\;\;\;\;\;\;\;\;\;\;\;\;\;\;\;\;if\;C\geq \theta_{2}\\ \gamma_{max}\frac{C-\theta_{1}}{\theta_{2}-\theta_{1}}\;\;if\;\theta_{1}\leq C<\theta_{2}\\ \;\;0\;\;\;\;\;\;\;\;\;\;\;\;\;\;\;\;\;\;\;\;\;\;\;\;\;if\;C<\theta_{1} \end{array}$$

In these mathematical functions, $\theta_{1}=0.2$ and $\theta_{2}=0.3$ are the thresholds for conjugation rate. The parameter $\gamma_{max}$ is the maximum value for conjugation rate (0.001, 0.01, 0.1 or 1), and *C* represents the available nutrients as explained above.

If conjugation occurs, this bacterium that received the plasmid will be called a transconjugant and have a fitness cost b (0.2, 0.4 or 0.6) for acquiring the plasmid. In this paper, we denote replicated transconjugants also as transconjugants. After a certain number of duplications (70 or 400), we assume that the bacterium has a compensatory mutation, and consequently, the cost reduces to the cost paid by donor cells (c).

Finally, if the selected bacterium is a donor or transconjugant, it can lose the plasmid with a probability 10^−3^, and if it does, it will be called segregant and will no longer suffer the fitness cost (because the plasmid is not there anymore). However, if the segregant bacterium receives the plasmid again, the same fitness cost reappears (the same value before losing the plasmid).

We select bacteria until 95% of the grid spaces are filled. When this occurs, we simulate the bacterial death of part of the population, leaving only 50% of the sites randomly filled. This process is repeated 1073 times or until there are only segregants and one more type of bacteria on the grid (donors, recipients, or transconjugants). Therefore, the computer model simulates 1000 bacterial generations on average for each combination of parameters and replicate.

### 2.3. Fitness Analysis

To calculate the relative success of type A cells compared to type B cells, we use the mathematical expression:

(3) SAB=Ln(NAfNAiNBfNBi)time=Ln(NAfNAi)−Ln(NBfNBi)time=mA−mB

In these mathematical expressions, the variables $N_{A_{f}}$, $N_{A_{i}}$, $N_{B_{f}}$, and $N_{B_{i}}$ correspond to the final and initial number of type A and B cells. If the density of type A cells increased relative to type B cells, then $S_{AB}>0$. If the opposite happens, $S_{AB}<0$.

To understand the impact of the transconjugants not transferring the plasmid to other cells, we calculated the difference between the relative success of the donors in the simulations, either allowing or not the transfer from transconjugants. Values below zero indicate that plasmid transfer from transconjugants contributes to the success of the donor population.

Statistical tests were performed in R v.3.5.1, available at http://www.rstudio.com/ (accessed on 23 February 2023) [25]. We performed triplicates of all simulated cases and performed one-sample Student t-tests (α = 0.05).

## 3. Results

Our previous work suggested that plasmid transfer and the fitness cost imposed on the recipient bacteria for receiving the plasmid can explain the success and maintenance of plasmid-bearing cells among bacterial populations. We considered that the ability of the transconjugant bacteria and donors to transfer the plasmid is equal. However, this assumption is usually not observed because the plasmid transfer rate is highly diverse, varying several orders in magnitude even among strains of the same species [20,26,27]—see introduction. Therefore, we compared the success of donor cells in the original scenario (in which transconjugants and donors transfer the plasmid with equal probability) with the scenario in which transconjugants cannot transfer the plasmid (the original donor cells and their descendants are the only ones that can transfer the plasmid to other cells). These two simulated scenarios would represent two disparate situations.

**Figure 1:**
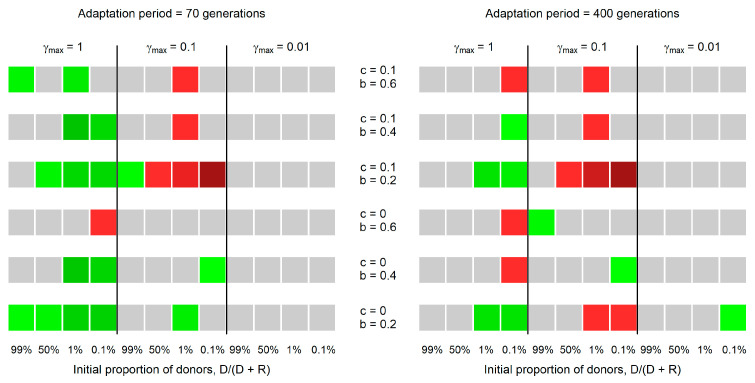
The difference in the relative success of donors considering that transconjugants cannot and can transfer the plasmid. Positive values (green squares) represent the cases where donors are more successful when the transconjugants cannot transfer the plasmid. Negative values (red squares) represent the cases where donors are less successful when the transconjugants can transfer the plasmid. Lighter colors represent values closer to zero, and darker colors represent values further from zero. Gray squares represent cases where the difference is not significantly different from zero (one-sample *t*-test, *p*-value < 0.05). Parameter c represents the fitness cost of the plasmid in donor cells, while b represents the fitness cost in unadapted transconjugants. Parameter *γ_max_* represents the maximum plasmid transfer rate from donors to plasmid-free cells (recipients or segregants). In the simulations where transconjugants can transfer the plasmid, *γ_max_* also represents the maximum transfer rate from transconjugants to plasmid-free cells (recipient cells or segregants). The transfer rate is at its maximum value if nutrients are abundant (see Equation (2)).

Figure 1 represents the difference in the success of the donor population in this new model (where transconjugants do not conjugate) and in the original model (where transconjugants and donors have the same ability to transfer the plasmid). To calculate the success in each case, we used Equation (3), where type-A cells are the donor cells and where type-B cells are the recipients, transconjugants, and segregant cells. The results show that the advantage to donors is higher if transconjugants do not pass the plasmid in some cases. This result occurs mainly when donors are rare at the beginning of the simulation and when the conjugation rate is high (Figure 1).

In the introduction, we suggested two hypotheses that might explain the advantage obtained by donors when the transconjugants do not pass the plasmid: (i) there are fewer transconjugants, hence fewer opportunities for compensatory mutations to appear, and (ii) donors are in greater numbers in the neighborhood of the conjugation events (hence close to these events).

To understand our first hypothesis (whether this advantage is related to the number of transconjugants that undergo the compensatory mutation), we compared the number of compensatory mutations in the model in which the transconjugants pass the plasmid with the model in which they do not.

First, let us focus on a case (c = 0; b = 0.4; $\gamma_{max}$ = 1) in which donors have an increased advantage when transconjugants do not transfer the plasmid compared to the case where transconjugants can pass the plasmid (with 1% donors and an adaptation period of 70). In this case, 47,979 compensatory mutations occurred throughout the simulation where transconjugants can transfer the plasmid, and 0 compensatory mutations occurred in the model where transconjugants cannot transfer the plasmid. This result shows that compensatory mutations among transconjugants are much more frequent in the model where transconjugants can transfer the plasmid (generating more transconjugants), suggesting that the lower number of opportunities for compensatory mutations among transconjugants contributes to the additional advantage to donors in the model where transconjugants cannot transfer the plasmid.

Now, we focus on a case (c = 0.1, b = 0.4, and $\gamma_{max}$ = 0.1) where donors have no further advantage when transconjugants cannot transfer the plasmid (also with 1% donors and an adaptation period of 70). In this case, there were no compensatory mutations in either model. Therefore, when compensatory mutations were already absent (in the model where transconjugants could transfer the plasmid), avoiding plasmid transfer from transconjugants conferred no further advantage to the donor population. This result corroborates the hypothesis that the number of opportunities for compensatory mutations contributes to the additional advantage to donors when transconjugants do not transfer plasmids.

To test our second hypothesis (that donors could benefit from conjugation events between transconjugants and recipient cells if they were near these conjugations, that is, if they were in the nutrient neighborhood of the recipient cell that receives the plasmid), we counted the number of each type of bacteria in the nutrient neighborhood of recipient cells receiving a plasmid. We also measured the distance of these recipient cells to the nearest donor cell. We have performed these computations for all conjugation events throughout the simulation. We compared two cases (both with adaptation time of 70 and 1% donors)—see Table 1. Note that the maximum number of cells in the nutrient neighborhood of any given cell is 7 × 7 − 1 = 48.

In the first case, the benefit to donors is lower when transconjugants do not transfer the plasmid than when they do (c = 0.1, b = 0.4, $\gamma_{max}$ = 0.1, and initial percentage of donors = 1%). In this case, (three left columns of Table 1), the mean number of transconjugant cells in the nutrient neighborhood of conjugation events was 1.76 lower in the case where transconjugants cannot transfer the plasmid than in the case where they can. The number of donor cells was only 0.29 higher. However, an additional 1.53 recipient cells are in the nutrient neighborhood of conjugation events than when transconjugants can transfer the plasmid. Thus, in the case of transfer from transconjugants, there are 15.26 recipient cells in the neighborhood of the conjugation events but one fewer donor cell, on average. Therefore, recipient cells have an increased advantage in the system where transconjugants do not transfer plasmids than in the system where they do.

We now analyze a different case, where donors have an additional advantage when transconjugants do not transfer the plasmid (c = 0, b = 0.4, and $\gamma_{max}$ = 1, and initial percentage of donors = 1%). In this case, (Table 1—three columns on the right), the number of recipient cells in the nutrient neighborhood of conjugation events changed from 5.35 to 7.03 on average. The number of transconjugants in the same neighborhood is much lower (less 14.65) when transconjugants do not transfer the plasmid (4.52 cells transconjugants) than when they do (19.17 transconjugants). Donors show the opposite trend, increasing substantially from 7.67 donor cells in the nutrient neighborhood in the system where transconjugants transfer the plasmid versus 20.79 donor cells in the system where transconjugants cannot transfer plasmids. Therefore, donor cells are the ones that benefit most from the conjugation events in the model where transconjugants do not transfer plasmids.

We also analyzed the distance of conjugation events to the nearest donor in the two cases and compared the two models. In the first case, the average distances were similar in the two systems: 2.17 and 1.89 in the systems where transconjugants can and cannot transfer plasmids, respectively. However, in the second case, the two distances are 27.69 and 1.43, respectively. Therefore, in this second case, the average distance between conjugation events and the nearest donor cell is much lower in the system where transconjugants can transfer the plasmid than in the other system. These shorter distances allow donor cells to benefit from conjugation events, contributing to explaining their higher advantage when transconjugants cannot transfer plasmids (green dot in Figure 1).

## 4. Discussion

Our results show that the selective advantage of the original donor population is higher if neither the transconjugants nor their descendants transfer the plasmid to other cells under three conditions. These conditions are: (i) the initial frequency of donor cells is low (i.e., when the initial values of D/(D + R) =1% or 0.1%), (ii) primarily for short adaptation periods (more common for an adaptation period of 70 generations than of 400 generations), and (iii) when the plasmid transfer rate from donors is high (i.e., when $\gamma_{max}$ = 1). We now explain the role of these conditions.

In the introduction, we mentioned two factors that could negatively impact the success of donor cells if transfers from transconjugants occur frequently. First, the higher the percentage of transconjugants, the higher the probability of the appearance of compensatory mutations in transconjugants and their descendants. This factor explains the conditions (i) and (ii) mentioned in the previous paragraph. Second, plasmid transfers from transconjugants to recipient cells far from donor cells would confer an advantage to transconjugant cells and not the original donor cells. This factor reinforces the importance of condition (i): the benefit to donors is higher if transconjugants do not transfer the plasmid, mostly when the proportion of donors is low because, in this case, donors would be far away from most transconjugants. In other words, if transconjugants and their descendants transfer the plasmid, donor cells would not benefit from transfer events. Finally, the third condition, high transfer rate, ensures that many transfer events occur, hence amplifying the difference between the two cases of allowing and not allowing plasmid transfer from transconjugants.

The conclusion that, at least in some conditions, the harmful impact of plasmid transfer would be higher if there were no transfers from transconjugants (or if these transfer rates were low) is remarkable for two main reasons. First, the idea that parasites or pathogenic agents (here, the plasmid) may play the role of biological weapons often relies on the assumption that these “weapons” replicate inside their victims, further increasing the number of “weapons” [28,29]. Second, Brown et al. [30] have argued that a fundamental difference between toxins and parasites as biological weapons is the ability of parasite amplification in their victims [30]. Therefore, it is interesting to note that if there are no transfers from transconjugants, then conjugative plasmids are, to some extent, playing the role of toxins “segregated” by donor cells, not biological weapons. The number of toxin molecules does not increase when they harm a victim. For example, the number of bacteriocin molecules does not increase when killing bacterial cells, and in contrast, the number of bacteriophages increases when they kill bacterial cells [28,30,31,32,33,34].

For both lysogenic bacteriophages and conjugative plasmids, the invasion rate into cells already occupied by a similar bacteriophage or plasmid (respectively) is several orders of magnitude lower than the rate of entering cells without these elements. Indeed, we can assume that there is no lysogenic bacteriophage entry nor plasmid transfer into cells already harboring a similar bacteriophage integrated into the chromosome or a similar plasmid, respectively.

A significant difference between conjugative plasmids and lysogenic bacteriophages is that most of the latter kill their hosts while plasmids do not. Therefore, it seems paradoxical to observe that although the cost imposed by plasmids is mild, the benefit to donor cells maintains or becomes even higher if transconjugants do not transfer the plasmid. This surprising conclusion deserves an explanation. For that, it is helpful to compare conjugative plasmids with temperate (lysogenic) bacteriophages in the context of this paper (biological weapons).

A primer on temperate bacteriophages is necessary. Non-temperate phages can only follow the lytic cycle (see below), but temperate phages may also follow the lysogenic cycle. Temperate bacteriophages are diverse, so let us focus on the lambda phage and the host *Escherichia coli.* For more information on bacteriophages, see [35,36,37,38,39].

Lambda phages may follow two different cycles: lytic or lysogenic. Consider a community of *E. coli* bacterial cells living in a habitat where one of the cells is a “lysogen”—an *E. coli* cell with a phage integrated into its chromosome. Whenever the bacterial cell replicates, phage genes replicate along with the bacterial genes. Therefore, if this cell replicates several times, forming a colony, all the descendant cells are “lysogens” because they all contain the lambda phage integrated into the chromosome. The other co-inhabiting cells in the community (without the lambda phage) may also replicate. Because they do not harbor the phage, they are not immune to the phage. Moreover, in principle, these cells do not have mutations conferring resistance to the lambda phage, so these cells are fully susceptible to the virus. Due to environmental causes such as UV light, a random lysogen initiates the lytic cycle. After half an hour, the cell bursts releasing around 100 phages ready to enter nearby susceptible cells.

Note that the other lysogens in the same colony, but not the other *E. coli* cells in the community, are immune to these phages. Therefore, the released lambda phages may infect nearby susceptible *E. coli* cells in the community. Many lambda phages follow the lysogenic cycle—integration into the bacterial chromosome—but many others follow the lytic cycle after entering the *E. coli* cells. The lytic cycle consists of virus replication several times inside the host, followed by the killing of the host and a burst of around 100 phages ready to infect other 100 cells, etc. In other words, there is an amplification of the number of phages in the habitat. The other lysogens remain alive and can use the resources left by all the killed susceptible cells. Therefore, the death of the other hundreds of cells belonging to different strains but competing for resources with the colony of lysogens (growing in the same habitat and/or feeding on the same nutrients) may largely counterbalance the single death of the original lysogen [28].

While phages may infect cells far from the original death cell, conjugative plasmids transfer directly from one cell to another—a phenomenon where the two cells need to be very close to each other for the plasmid to be able to transfer. Therefore, only the plasmid-donor and a few more cells very close to the conjugation event can use the resources not used by the cell that received the plasmid. Although the impact on recipient cells is low (note that plasmids do not kill cells), the unused resources are only available locally.

The significant differences between the two mobile genetic elements (temperate phages versus conjugative plasmids) are the following (in the context of biological weapons). First, with burst sizes as big as 100, phages can harm many more competitors of the original lysogenic population than conjugative plasmids, which harm only a single victim recipient cell in a similar period. Second, while plasmid donors are almost the only cells taking advantage of the resources unused by their victims, the thousands or millions of cells present at the colony of lysogens must share the unused resources, not only among them but also with many other cells in the same habitat (including cells belonging to other species, mostly unaffected by the lambda virus). Therefore, in the case of this paper, where we analyzed the impact on donor cells of plasmid transfer from transconjugants, one can understand why the benefit for donor cells is higher if transconjugants do not transfer the plasmid in some conditions. After a few successive transfer events, donor cells are too far away from unused resources to benefit from it.

Plasmids are highly diverse, but, arguably, bacteriophages are even more diverse. For example, there are double-stranded and single-stranded DNA bacteriophages as well as RNA-based bacteriophages [35,36]. The previous paragraphs discussed the differences between plasmids and bacteriophages. However, some of these elements, denominated phage-plasmids, are both bacteriophages and plasmids [40]. Future work should study the effect of these elements as biological weapons.

This work shows the importance of plasmid transfer in the context of social behavior between bacteria. It is well known that beneficial genes, such as antibiotic resistance genes, often carried by conjugative plasmids, are responsible for maintaining these DNA elements among bacterial populations [41]. Other works have suggested that some plasmids “behave” as parasites because they compensate for the fitness cost imposed on their hosts by spreading efficiently between hosts [42]. Our previous work [17] proposed that plasmid transfer may help donor cells compete for resources with other cells in the same habitat, and this paper proposes that the benefit to donor cells is even higher if transconjugants do not transfer the plasmid in some conditions. This result strengthens the hypothesis of plasmids as biological weapons because previous studies have shown that most bacterial cells are poor plasmid donors [20,26,27], which may imply that most transconjugants have a low ability to transfer their plasmid to other cells.

We now consider the limitations and strengths of this study and the underlined hypothesis.

(i) We always considered that compensatory mutations occurred in chromosomes. In another work submitted elsewhere [43], we study the validity of this hypothesis when compensatory mutations occur in plasmids. To our surprise, the effect is almost the same irrespective of the replicon where compensatory mutations occur. The two main reasons are that the period before compensatory mutations occur is the most significant and that transfer events of mutated plasmids usually occur in sites far from the original donors.
(ii) Both plasmids and chromosomes may evolve, and we have already considered the appearance of compensatory mutations, but other mutations may occur during the 1000 bacterial generations considered in this study. These mutations may also help plasmids to survive. For example, plasmids may have the opportunity to receive antibiotic-resistance genes in a transposon that originates from the chromosome or another plasmid present in the same cell. However, plasmids must survive while these genes, compensatory mutations in the plasmid itself or chromosome, or other types do not arise (for example, genes encoding for H-NS proteins that silence horizontally acquired DNA with an AT content higher than that of the chromosome [44,45]). The model/mechanism considered here and elsewhere [17,43] gives plasmids the necessary conditions and time to wait for those advantageous changes. Moreover, plasmids sometimes confer no cost [27], so their maintenance deserves no more explanations. In these cases where the cost is null in a specific environment, there is no guarantee that the plasmid is also costless in other environments; if it is costly in other environments, some mechanism must ensure and explain their maintenance, e.g., the one discussed here or in [46].
(iii) We assumed that the plasmid transfer rate did not change. Bacterial conjugation is often tightly regulated: plasmids frequently encode genes whose function is to decrease their transfer rate. Plasmids in which these genes are mutated transfer more efficiently than the wild-type plasmid. For example, the IncFII R1drd19 plasmid is the corresponding derepressed mutant of the wild-type (repressed) R1 plasmid [47], and the transfer rate between *Escherichia coli* K12 MG16555 cells of the former plasmid is about 1000 times higher than that of the latter plasmid [20]. Repressed plasmids express lower levels of sex-pili, becoming less susceptible to the so-called male-specific-phages: bacteriophages that infect bacteria precisely through these sex-pili, a hypothesis first proposed by Anderson already in 1968 [48] (see also a refinement of this hypothesis in [49]). Later Lundquist and Levin found that some plasmids become transitory derepressed after a few transfers [50]. In principle, transitory derepression would weaken the hypothesis of the present study because, with transitory derepression, many more transfer events would occur further away from the original donor cells. Some genes are involved in transitory derepression (e.g., the products of the *finO* and *finP* genes repress conjugation). However, one cannot be sure that transitory derepression would work in different strains. Moreover, as argued above, the hypothesis discussed in the present study aims to explain how plasmids are maintained before these other genes arise or while these systems are not helpful.
(iv) A limitation of this study is that it is based on a mathematical model [17] and computer simulations ([17] and the present study), rather than on experimental results. We base our simulations on previous studies that have constructed and adjusted the computer model and its parameters to ensure that computer results match those obtained in laboratory experiments [22,23,24]. Moreover and most importantly, simulations allowed us to test hundreds of different conditions and parameters, putatively simulating different bacteria and plasmid types.

## 5. Conclusions

In a previous work [17], we have shown that donor cells may increase their relative fitness by interference competition due to the transfer of their plasmid into neighboring cells. Therefore, plasmid-bearing cells may use their conjugative plasmids as biological weapons. Contrary to the primary hypothesis of biological weapons, where contagion between victims increases the negative effect of the weapon, the results presented here show that if there are no transfers from transconjugants to other cells, the benefit to the original donor population may be even higher than if transconjugants can transfer the plasmid. Because most bacteria transfer conjugative plasmids inefficiently [20,27], these results strengthen our original hypothesis that plasmids may maintain among bacterial populations by conferring advantage to their hosts as biological weapons. With this process, conjugative plasmids may secure their maintenance; later, they can integrate host-beneficial genes, such as virulence or antibiotic resistance, into their DNA, giving additional advantages to plasmid-bearing cells [51,52], or even genes that could decrease plasmid cost (e.g., coding for the H-NS protein) or increasing transfer rate.

## Figures and Tables

**Table 1:** The average number of each bacterial type in the nutrient neighborhood and the distance of conjugation events to the nearest donor throughout the simulation.

	c = 0.1; b = 0.4; $\gamma_{max}$ = 0.1 *			c = 0; b = 0.4; $\gamma_{max}$ = 1		
Transfer from transconjugants?	Yes	No	Difference	Yes	No	Difference
Recipients	13.73	15.26	1.53	5.35	7.03	1.68
Donors	13.98	14.27	0.29	7.67	20.79	13.12
Transconjugants	4.29	2.53	−1.76	19.17	4.52	−14.65
Adapted transconjugants	0	0	0	6.43	0	−6.43
Segregants	0.32	0.27	−0.05	0.08	0.11	0.03
Distance to nearest donor	2.17	1.89	−0.28	27.69	1.43	−26.26

* Parameters c and b represent the fitness cost of the plasmid in donor cells and unadapted transconjugants. The parameter $\gamma_{max}$ represents the plasmid transfer rate.

## Data Availability

Code and data are available on GitHub (https://github.com/jrebelo27/harmfull_plasmids_no_re-transfer accessed on 25 April 2023).
